# Immune-neuroendocrine reactivity and features of tuberculosis in pregnancy

**DOI:** 10.3389/fmed.2025.1503402

**Published:** 2025-02-05

**Authors:** Anna Starshinova, Leonid P. Churilov, Igor Kudryavtsev, Artem Rubinstein, Ekaterina Belyaeva, Anastasia Kulpina, Hong Ling, Min Zhuang, Dmitry Kudlay

**Affiliations:** ^1^Saint Petersburg State University, Saint Petersburg, Russia; ^2^Almazov National Medical Research Centre, Saint Petersburg, Russia; ^3^Department of Immunology, Institution of Experimental Medicine, Saint Petersburg, Russia; ^4^Harbin Medical University, Harbin, China; ^5^I.M. Sechenov First Moscow State Medical University, Moscow, Russia; ^6^Institute of Immunology FMBA of Russia, Moscow, Russia; ^7^Lomonosov Moscow State University, Moscow, Russia

**Keywords:** tuberculosis, TB/HIV, coinfection in pregnant women, immune response, neuroendocrine reactivity

## Abstract

The combination of tuberculosis and pregnancy always raises questions about therapy, the specialness of management of pregnancy, obstetrics, postpartum period, and lactation; the effect of therapy on fetal development and the peculiarities of the tuberculosis course. Until recently, tuberculosis and pregnancy were considered a rare combination, but with the growing problem of HIV infection and worsening tuberculosis screening among adults, this combination has become quite common. Moreover, cases of congenital tuberculosis in newborns have begun to emerge. In this review, we analyzed features of immunologic and immuno-neuroendocrine reactivity in pregnant women that influence for prevalence TB and TB/HIV coinfection. The immuno-neuroendocrine changes characteristic of pregnancy have a multifactorial effect on antituberculosis immunity and determine the specificity of the course of tuberculosis against the background of pregnancy. These changes contribute to a more severe course of TB than before pregnancy. The structure of TB clinical forms in women who became ill during pregnancy and in the first year after childbirth is characterized by greater severity, higher frequency of multi-organ lesions, and the percentage of bacterial isolates is significantly higher among women with TB that developed in the postpartum period compared to women who developed it during pregnancy. HIV infection poses a particular threat, exacerbating immune response disorders that affect the effectiveness of treatment and disease progression in general.

## 1 Introduction

Nowadays, tuberculosis continues to be a significant problem that mankind has not yet been able to solve. Among infectious diseases, tuberculosis (TB) still is the most globally significant as a leading cause of death worldwide. According to the World Health Organization (WHO), nearly 10.6 million new TB cases (range 9.9–11.4 million) were identified in 2022, indicating an increase of 3.5% from the reported 10.3 million (range 9.6–11.0 million) in 2021. The incidence of TB increased by 3.9% from 2020 to 2022. In 2022, TB was a cause of 1.3 million deaths globally, the same level as reported in 2019. In 2023, WHO reported an increase in the number of new cases of tuberculosis and an increase in the number of new deaths caused by this infection (1, 2).

A new coronavirus infection (COVID-19) has significantly impacted the well-established monitoring measures and tuberculosis support programs in many countries around the world (3).

According to a mathematical analysis in one of the studies, the authors calculated that during the spread of COVID-19, mortality from HIV, tuberculosis, and malaria may increase by 10, 20, and 36%, respectively, over the next 5 years (4).

Predictions about the impact of the COVID-19 pandemic on tuberculosis morbidity and mortality in high-burden countries have been fulfilled (2). However, thanks to the adopted programs and governmental support, introduction of new technologies for diagnostics and treatment of tuberculosis infection, monitoring of epidemic indicators and formation of new guiding documents, the Russian Federation has achieved stabilization of the epidemic situation and systematic reduction of tuberculosis morbidity and mortality rates by several times (5). In 2021, the Russian Federation was removed from the list of countries with a high burden of tuberculosis infection, but remained on the list of countries with a high burden of multidrug-resistant tuberculosis, including the cases combined with HIV infection (2).

Tuberculosis in combination with HIV infection is particularly dangerous due to rapid generalization of infection against the background of immunosuppression and low efficiency of treatment in patients with combined pathology (6, 7).

Of particular concern are those countries with a high burden of tuberculosis, where high levels of epidemic tension may develop in the absence of adequate interventions to detect new cases of the disease (8). Maternal death, termination of pregnancy, preterm delivery and low birth weight of the newborn were the most common adverse outcomes in pregnant TB patients, especially those with drug-resistant pathogens (9). According to the studies, the maternal mortality in pregnant TB patients was 7.5% (95% CI 3.2–12.8), 10.6% (95% CI 6.0–16.3) for pregnancy loss, 12.9% (95% CI 0.0–38.0) for preterm delivery and 23.7% (95% CI 17.0–31.0) of the cases had low birth weight of the baby (10).

There are no clear statistics on the prevalence of tuberculosis among pregnant women, but some studies have reported rates ranging from 0.06 to 0.25% in low-prevalence countries and 0.07–0.5% in high-prevalence countries, increasing to 0.7% with HIV co-infection (11, 12). Tuberculosis is often co-infected with other specific infections: not only HIV infection (15%), but syphilis (10%), and viral hepatitis (4%)—as well (9, 10).

Early diagnosis and treatment of active disease can reduce maternal and neonatal morbidity and mortality. Increased use of new rapid molecular assays with drug susceptibility testing has significantly shortened the process of diagnosing active tuberculosis (12). Undoubtedly, early diagnosis and treatment of active tuberculosis can reduce maternal and neonatal morbidity and mortality. The widespread application of new rapid molecular assays testing for drug resistance of *Mycobacteria* will allow adequate treatment of pregnant women (13).

The combination of tuberculosis and pregnancy always raises questions about the prescription of therapy, the specialness of management of pregnancy, obstetrics, postpartum period, and lactation; the effect of therapy on fetal development and the peculiarities of the tuberculosis course (14). Until recently, tuberculosis and pregnancy were considered a rare combination, but with the growing problem of HIV infection and worsening tuberculosis screening among adults, this combination has become quite common. Moreover, cases of congenital tuberculosis in newborns have begun to emerge (15).

Basing on analysis of data from more than 57 million pregnant women and birth information, HIV-TB was associated with the highest risk of several obstetric complications, as well as with alcohol and drug abuse. Mothers with HIV-TB coinfection have a high risk of obstetric complications (11, 12). According to many studies, pregnant women with HIV infection and tuberculosis are 2.5 times more likely to transmit the virus to their children. This category of pregnant women has 2–3 times higher mortality and 3–4 times higher infant mortality compared to HIV-negative pregnant women (6). Children born to pregnant women with tuberculosis and HIV are small-for-date born and are 24 times more likely to develop tuberculosis. Preventive treatment is of particular importance, especially in low content of CD 4+ lymphocytes (12, 15).

In the present context, it is particularly important to systematize existing knowledge and current data on the management of pregnant women with suspected and diagnosed tuberculosis infection. The comprehension of the lessons learned is relevant not only for expectant mothers but also for newborns.

## 2 Impact of pregnancy on the tuberculosis course

Pregnancy and tuberculosis (TB) is an unfavorable combination for women, despite conflicting views among researchers. The impact of TB on pregnancy may depend on many factors, including the severity of the disease, gestational age at diagnosis, HIV co-infection, and the quality of treatment (16, 17).

According to domestic researchers, the risk factors for worsening the course of TB during pregnancy and its progression in the postpartum period are endocrine restructuring, blood loss, lactation, emotional stress, inadequate treatment or complete absence of TB treatment during pregnancy (18). In addition, the development and progression of TB during pregnancy and after childbirth are caused not only by the listed medical and biological, but also by some social factors: low socio-economic standards of living, poor and/or insufficient nutrition (nutritional deficiency of proteins, carbohydrates, and essential nutrients), unfavorable housing conditions, family relationships, social instability (loneliness, lack of marriage), tobacco smoking, alcohol abuse, and drug use (13, 19).

In the second half of pregnancy, as a result of significant uterine enlargement, the ratios in the volumes of abdominal and thoracic cavities change, there persists a high standing of the diaphragm, which seems to mimic the therapeutic effect of pneumoperitoneum. Immediately after delivery, as a result of a sharp phrenic muscle lowering, the so-called “abdominal decompression” occurs, which promotes aspiration of caseous masses into healthy lung tissues and contributes to bronchogenic infiltration (13). In addition, the hypertension developing during labor in the small circulation circle creates a threat of pulmonary hemorrhage and spontaneous pneumothorax (20).

Severe preeclampsia, eclampsia, *placenta previa*, postpartum hemorrhage, sepsis and anemia were more common among mothers diagnosed with TB (21). In addition, negative consequences of the disease and its treatment for the child: congenital malformations in the fetus in the form of shortening of the tubular bones, dysplasia, and incontinence of the soft palate have been identified (22).

The results of some studies have shown that the presence of concomitant pathology in pregnant women, including cardiovascular disease, anemia, diabetes mellitus, etc., leads to complications in the postpartum period and, in 63% of cases, to progression of tuberculosis infection (20, 21). Several studies have shown that the hormonal and immunologic changes occurring in women during pregnancy lead to a decrease in the proinflammatory activity of CD4+ lymphocytes, which leads to increased susceptibility to infection, TB reactivation, and causes its low-symptomatic course (13, 19). These changes can explain the higher risk of progression of active TB in women both after childbirth and after induced termination of pregnancy (2–2.7 times higher than in non-pregnant women) (23).

## 3 Peculiarities of immune response in pregnant women against the background of tuberculosis infection

The effective immunologic response to *M. tuberculosis* antigens involves predominantly type 1 immune reactions, with IFNγ-producing CD4+ and CD8+ T cells being the main contributors (24, 25). Also, the interaction between macrophages and Th1 plays a major role in limiting the spread of tuberculosis infection (26).

During pregnancy, there is a shift in immune response from cell-mediated (Th1) to antibody-mediated (Th2) immunity. The role of immunity in the success of pregnancy is undoubtedly more complex. It includes Treg cells with specificity to allogeneic antigens expressed by the fetus and NK cells, HLA-G and macrophages and dendritic cells, reduced levels of the pro-inflammatory cytokine IL-17 may also contribute to the likelihood of developing TB (20).

The same cells are involved in the delayed-type hypersensitivity reactions contributing to the formation of tuberculosis granulomas, and thereby limiting the spread of the pathogen (27). Type 2 immune response, on the contrary, contributes to the suppression of the immune response to tuberculosis infection, since the main cells involved in this type of immune response are Th2 cells, which have an anti-inflammatory cytokine profile (26). These cells are able to inhibition of Th1 differentiation, which may promote the spread of the pathogen (28).

Thus, the main cells providing effective defense of the organism against tuberculosis infection are macrophages, IFNγ-producing CD4+ and CD8+ T cells. Various immunodeficiency conditions (such as HIV infection, diabetes mellitus, starvation, chronic kidney disease, etc.), as well as a shift in immunologic balance toward an anti-inflammatory profile contribute to immunodepressive state and suppression of these cells, which can lead to reactivation of latent tuberculosis infection (LTBI) and worsening of the course of active tuberculosis (TB). Numerous studies have shown that pregnancy is a physiologic cause of relative immunе suppression (29). Down regulation of immunity in pregnancy, as well as a shift in immunologic balance toward regulatory immunity, is necessary to prevent fetal rejection (30–32). Consequently, pregnancy shifts the immune response toward an anti-inflammatory profile, which may suppress macrophages and IFNγ-producing Th1, CD8+ T cells and lead to impaired immunologic control of *M. tuberculosis*.

Birku et al. showed that pregnant women with LTBI have reduced numbers of *M. tuberculosis*-specific IFNγ-secreting cells (33). Similarly, pregnant women co-infected with HIV and LTBI had a reduced IFNγ-associated *M. tuberculosis*-specific cellular response compared to non-pregnant women with verified HIV and LTBI co-infection. Another team of authors obtained analogous results (34).

Ranaivomanana et al. in their study examined pregnant women with both latent and active tuberculosis infection. It was found that IFNγ production by CD4+ and CD8+ T cells was elevated in the group of pregnant women with LTBI compared to pregnant women with ATB. Moreover, when pregnant women with latent tuberculosis infection were compared with non-pregnant women with LTBI, greater IFNγ CD4+ T-cell production was observed in the latter group (34). Thus, it can be concluded that pregnancy suppresses the *M. tuberculosis*-specific IFNγ-associated cellular response in women with LTBI.

It is worth noting that the level of IFNγ response to *M. tuberculosis* antigens varies depending on the stage of pregnancy. For example, during labor, the lowest IFNγ response to Mtb antigens is observed (35). Indeed, the balance of pro- and anti-inflammatory immune responses changes depending on the trimesters of pregnancy.

By the third trimester, the concentration of estradiol, estriol and progesterone gradually increases, while at the same time a shift in the balance toward a predominance of anti-inflammatory phenotypes of immune system cells is noted. The point is that all these three hormones contribute to the suppression of differentiation and proliferation of CD4+ and CD8+ IFNγ-secreting cells, which are essential for the containment of tuberculosis infection (36–38).

In turn, under the influence of estradiol, estriol and progesterone, an anti-inflammatory profile of the immune response is formed (39, 40). For example, progesterone promotes the formation of IL-4 producing Th2 (41), and estradiol promotes the expansion of T cells with a regulatory phenotype of CD4 + CD25+. The greater level of estrogenes in postmenopausal women was observed in tuberculosis compared to healthy controls (42).

Recent studies have demonstrated a decrease in *M. tuberculosis*-specific CD4+ T-cell responses in the third trimester of pregnancy compared to the first and second trimesters, as well as to the pre- and postnatal periods (43). These changes mainly occurred in the effector memory compartment with CCR7-CD45RA- phenotype. Saha et al. also demonstrated nonspecific activation of T cells with HLA-DR + CD38 + CD4+ phenotype in interferon-gamma (IFNγ) release assay (IGRA)-positive women after delivery compared to IGRA-negative women (43). Nonspecific activation of T cells in latent tuberculosis infection may be regarded as a marker of tuberculosis process reactivation (44, 45). These findings suggest a decrease in selective Mtb-specific adaptive immune response, predominantly in late pregnancy.

Thus, immune responses mediated by IFNγ-producing CD4+ Th1 cells, macrophages and cytotoxic CD8+ T cells are associated with the control of bacterial replication and create an effective response to *M. tuberculosis* antigens, whereas T cells with a regulatory phenotype and CD4+ Th2 cells counteract these effects and contribute to the spread of infection. The effective immune response to *M. tuberculosis* in pregnant women is reduced due to physiologic mechanisms of immunosuppression and also depending on the presence of a comorbid background of the pregnant woman (e.g., HIV infection) (46, 47).

Based on the detection of Mtb-specific immune response, it is possible to predict reactivation of the tuberculosis process in pregnant women. Recent transcriptome studies have shown that in IGRA+ pregnant women, expression of ITSN1 and LONRF1 genes may be a predictor of postpartum tuberculosis reactivation. Among these genes, ITSN1 is responsible for CD4+ T-cell activation and promotes IFNγ production in response to *M. tuberculosis* antigens (48).

## 4 Immuno-neuroendocrine reactivity in pregnant women

Pregnancy in fact is the formation of a superorganism consisting of mother and fetus. It is characterized by a special state of the immuno-neuroendocrine apparatus favoring physiological productive inflammation and feto-maternal immunological interactions, which establish the successful placentogenesis (49).

Pregnancy as a phenomenon is surrounded in human culture by a high life-affirming pathos. The inertia of this general humanistic and even poetic attitude toward pregnancy has contributed to the long-held, but ungrounded notion in clinical science reasoning that the normal development of the mother-fetus relationship must be free of any immunologic conflicts (21, 50).

However, the biological side of pregnancy is simply prosaic. The fetus is a semi-allogeneic “allograft” (and in surrogate pregnancies it is completely xenogeneic), containing antigens inherited from the father that are alien to the mother’s body (51).

Back in the 1980s, W. P. Falk and P. M. Johnson convincingly showed that the features of the maternal immune system response to fetal antigens largely resemble the response to parasite antigens, and, apparently, the immunoneuroendocrine support of pregnancy in placental mammals was formed on the basis of phylogenetic modification of the antiparasitic immunity archetypes. The whole complex of mechanisms of fertilization, implantation and placentation emerged as a superstructure over the mechanisms of immunity, inflammation and, in particular, antiparasitic defense (50, 52). The maternal organism needs mechanisms that selectively weaken the immune attack against fetal antigens, but does not completely stop the immune response to the fetus. Thus, parthenogenetic development of oocytes in humans stops because of the absence of paternal antigens of the fetus as a basis for the immune response of the mother at the time when the placenta should begin to form. In humans’ parthenogenesis is just a infrequent cause of ovarian teratomas although it is observed as a routine phenomenon in rather complex vertebrate non-placental animals (49, 53, 54).

Placentogenesis is impaired in marriages of partners who are very close or match in major histocompatibility complex (MHC) proteins. This suggests that a moderate degree of maternal and fetal antigenic foreignness is necessary for controlled feto-maternal conflict. In this regard, placental formation itself, paradoxically, should be regarded as a phylogenetic modification of productive inflammatory mechanisms (49). Similar view was expressed in the egregious title of the article “The placenta is just a neuroendocrine parasite” by Lowry (55).

In order to ensure a moderately regulated response to the fetus leading to effective placentogenesis, both T-dependent immunologic reactions and manifestations of reagin-dependent immunity are enhanced during physiologic pregnancy.

### 4.1 Th2-mediated immunity and pregnancy

According to J. Horton and N. Ratcliffe, only placental mammals have IgE. In females of higher mammals, relatively higher titers of IgE are registered during pregnancy (40, 50).

The embryo is formed in conditions favorable for the development of Th2-dependent anaphylactic reactions: Thus, during pregnancy, the uterus, initially rich in mastocytes, contains even more of them; they are especially abundant near the embryo. The placenta secretes the anaphylaxis mediator histamine into the mother’s blood. Local anaphylactic reactions occur at the maternal-embryo tissue interface, leading to the formation of microthrombi and fibrinoid that partially shield embryonic antigens from the maternal immune system (56, 57). The placenta, in parallel with histamine hyperproduction, sharply increases histaminase activity, which protects the mother from the negative effects of hyperhistaminemia. This is a typical mechanism, which does not allow local and systemic regulatory effects to conflict during normal course of pregnancy.

I.I. Mechnikov was the first to postulate that the main function of the immune system is the formation and maintenance of body multicellularity and called such constructive reactions at the early stages of ontogenesis “physiological inflammation,” representing in his opinion “the struggle for existence between different cells of the body” (57). Developing the ideas of Mechnikov, some authors interpreted the very mission of immune system as “integrity protection,” not only by dangerous pathogens attacking, but also by support and protection of safe and useful commensals “conversion of parasitism into commensalism” by means of regulatory lymphocytes (58).

However, in granulomatous productive inflammation, characteristic of the tuberculosis pathogenesis, Th1-dependent mechanisms of immune response are quite essential, and the production of IgE reagins, on the contrary, is prognostically unfavorable (59). This sets the stage for the mutual untoward influence of tuberculosis and pregnancy.

### 4.2 Placental products, tolerance to fetus and potential immunosuppression

The placenta is known to be a polyhormonal, endo- and paracrine organ, the main conductor of the entire course of pregnancy. An important prerequisite for maternal tolerance of the fetus as an allograft is the unique features of the trophoblast, whose fetal cells contacting with the maternal body do not express polymorphic HLA molecules of either classes I or II. Only HLA-G molecules devoid of polymorphism are expressed (60).

Thus, the trophoblast does not elicit rejection reactions from T cells, and the non-polymorphic antigens of HLA-G block the KIR-receptors of maternal natural killer cells and prevent them from attacking the fetus. Maternal T cells are tolerant to HLA-G because her thymus epithelium expresses these proteins. Dendrocytes that could, by expressing HLA proteins, present antigens, are virtually absent in the normal placenta (49).

The placenta also secretes a number of immunosuppressive signaling molecules that significantly modulate immune reactions and inflammation, which cannot but affect the course of tuberculosis in pregnancy. Modification of a number of placental products occurs, giving them new immunosuppressive potencies. A placenta is not innervated at all, but nevertheless is capable of secreting a number of neurohormones. It is a source of a huge amount of corticoliberin, which is necessary to optimize its own microcirculation, but this does not lead in the pregnant person to severe chronic stress and hypercortisolism, although some cushingoid manifestations, such as striae in pregnant women, due to this still occur (61, 62). The reason is the protective anti-stressor effect of corticoliberin-binding protein produced by the liver and other cells in the body of pregnant woman. Due to this strong local effect of placental corticoliberin is accompanied just by weak systemic effect (55, 63).

At the same time, in the normal course of pregnancy, the developing fetus does not generate danger signals that could, due to the induction of costimulatory molecules on immune cells, enhance the immune responses; therefore, it is not damaged by the immune system, despite its obvious “foreignness,” according to the “danger hypothesis” of Matzinger (64).

When parents are close to each other in terms of HLA antigens, but also in case of pronounced immunosuppression in the mother (for example, during the development of tuberculosis combined with HIV infection), the percentage of miscarriages increases sharply (12). That is, normal pregnancy should be ensured by immune recognition of the fetus by the mother and their moderately regulated isoimmune interaction. Regulating it, the placenta incretes into the systemic bloodstream various peptide hormones (chorionic gonadotropin (CG), placental lactogen, corticotropin, chorionic thyrotropin, gonadoliberin, corticoliberin, tachykinins, etc.); enzymes (alkaline placental phosphatase, oxytocinase, histaminase, aromatase, etc.) and so-called trophoblastic “pregnancy zone proteins.” Several bioregulators are particularly significant, including trophoblastic β1-glycoprotein (TBG), CG, and *α*-fetoprotein (AFP) (65). The dominant protein secreted by the trophoblast and even the blastocyst (based on paternal sperm-supplied m-RNA) is TBG, discovered by domestic scientists as early as the beginning of 1970s and now known as PSG1 (or pregnancy-specific glycoprotein 1), the earliest immune regulator expressed in pregnancy (66).

TBG serves as a powerful immunomodulator, stimulating primarily T regulatory lymphocytes (Treg), which play an extremely important role in the formation of immune tolerance in general and to antigens of the fetoplacental complex in particular. As a result, their content increases during successful pregnancy. TBG both directly (67, 68) and indirectly through transforming growth factor β1 stimulates the differentiation of T lymphocytes into Treg. In relation to TB, this bioregulator, purportedly, may contribute to the latent course of TB observed in many pregnant women, which peculiarity is hardening the diagnosis (69).

Chorionic gonadotropin (CG) was discovered first among placental hormones in the urine of pregnant women back in 1927 (70), and its syncytiotrophoblastic origin was first shown by S.S. Chalatov, a graduate of St. Petersburg University, who in the early 1930s proposed the first therapeutic preparation of CG for pregnancy preservation (Leukozol^©^) (71). CG present in all cell membranes of embryonic and fetal cells and spermatozoa. Membrane-associated CG turns cells into immunologically unresponsive ones, with the same mechanism acting to elude neoplasia from immune surveillance (72, 73). In fact, this ligand is a one more “checkpoint” of the immune response, akin to the known T-cell inhibitory regulators CTLA and PD (74).

A wide range of complex progravidar and fetotropic effects of CG includes the effect on immunocompetent cells. Thus, Treg express LH/CG receptor and CG promotes their attraction to the uterus and placenta (75, 76). CG promotes the differentiation of T-lymphocytes into Treg, increasing their functional activity (77, 78). It can be assumed that CG in pregnancy does not favor anti-mycobacterial immunity, rather the opposite (79). Thus, there are old, but relevant observations that CG in experiment aggravated the course of lung and genital tuberculosis (80, 81). Interestingly, the substances have been found in the lysates of *Mycobacterium tuberculosis* lysates that block the CG-dependent stimulation of progesterone production (82, 83), thus making possible the antigonadotropic effect of tuberculosis in pregnancy, which indicates the negative effect of tuberculosis on its course (84, 85). Moreover, it has been shown that highly virulent strains of *M. tuberculosis*, in contrast to low-virulence ones, produce bacterial analogs of CG, apparently in an attempt to weaken the host immune response. Finally, CG in pregnancy enhances phagocytic functions of neutrophils, important participants of inflammation in tuberculosis foci (86).

A cytokine *α*-fetoprotein (AFP) is the main embryo-specific and tumor-associated protein of all mammals, actively produced during pregnancy. It stimulates the production of anti-inflammatory IL-10 by lymphocytes (in tuberculosis as well), and its production increases significantly in the latter (75). It even causes difficulties in differential diagnostics of tuberculous and neoplastic foci (87). Based on the data on the downward action of AFP in relation to immunoinflammatory reactions, it is considered to be one of the fetal defenses against the pathogenic action of the maternal immune system (88). According to Kemona et al. (89), it also stimulates incomplete phagocytosis by thrombocytes. Apparently, these aspects of AFP action do not contribute to active eradication of tuberculosis infection in pregnant women.

A particularly striking evidence of the homology of placental mechanisms of suppression of immune reactions in pregnancy and mechanisms of parasite impact on immunity are the facts established in the 21st century and related to the role of placental tachykinins and their phospholipid derivatives. Syncytiotrophoblast is capable of synthesizing neurokinin B, which besides placenta is expressed in brain only (90, 91). Synthesis of this peptide by the placenta occurs in normal pregnancy but increases many times in preeclampsia. Moreover, its exogenously administered excessive doses reproduce preeclampsia in experimental pregnant female rats (92).

This neurokinin has been shown to redistribute blood flow resources in the body in favor of the placenta and uterus, while inducing systemic arterial hypertension and tachycardia typical of preeclampsia. Its toxic concentrations cause systemic disruption of endothelial properties, disorder of hemostasis, altering platelets, neurons and contributing to nephropathy as well as seizures seen in eclampsia. They increase vascular permeability, especially in the small circle, which contributes to edemas, particularly pulmonary one (93).

The typical pathoinformational conflict of local and systemic regulation is reproduced, when the paracrine regulator in excess begins to disrupt the systemic neuroendocrine regulation of vital functions of the organism (94). The signal causing hyperproduction of neurokinin B by the placenta in preeclampsia is relative ischemia in the placenta due to insufficient implantation, which is typical for the etiology of preeclamptic conditions, including tuberculosis in pregnancy (95, 96).

This pathogenetic mechanism is analogous to the development of hyperreninemic renal arterial hypertension, when the ischemic kidney (in the struggle for its own blood supply) causes chronic increase of arterial blood pressure to the detriment of the body.

Moreover, placental neurokinin B, unlike brain neurokinin B, was found to be bound to a phosphocholine residue. Phosphocholine is an intermediate in the synthesis of phosphorylcholine and lecithin, it is plentiful in oocyte and placenta, being a ligand of C-reactive protein. A number of other placental neurohormones have also been found to be phosphocholinylated. Immunologically privileged placenta and testes are the only human organs where significant activity of the corresponding enzyme responsible for this modification of peptides was found: namely phosphocholine-cytidylyltransferase (97).

The enzyme is also expressed in tumors that escape immune surveillance. It is particularly interesting that phosphocholination is also used by round parasitic worms, *Filariae,* to impart immunosuppressive properties to the glycoproteins they secrete and to suppress the host immune response to parasites (98, 99). Again, this fact emphasizes the analogies between immunity in pregnancy and in parasitoses (59).

Phosphocholinylated peptides shift the direction of differentiation of T-helper precursors toward Th2, suppressing the development of Th1 and Th17, promoting anaphylactic processes (which, as mentioned above, are associated with placentogenesis) and preventing the immune response to endocellularly presented antigens, including fetal ones, but also mycobacterial antigens! The mutually aggravating course of helminthic diseases, especially opisthorchiasis, and tuberculosis is well-known (100). There is evidence of weakening of cellular immunity, in particular, the functions of cytotoxic T-lymphocytes in filariasis (101). However, if essential elements of antituberculosis defense are affected by phosphocholinylated peptides in helminthiasis, this may also occur in pregnancy accompanied by their hyperproduction, especially in preeclamptic states.

Interestingly, pregnancy alleviates the course of some autoimmune diseases in which phosphorylcholine peptides have been successfully used in experimental therapy, for example, rheumatoid arthritis (102), and such clinical observations led to a number of attempts to use placental extracts and blood serum of pregnant women in rheumatoid arthritis and in autoimmune eye diseases as early as the late 1940s and early 1950s: first in the USSR (by V. P. Filatov and his school), and later also in other countries (102–105).

Tuberculosis is closely associated with preeclamptic and eclamptic complications of pregnancy, which is not coincidental but apparently reflects the effect of the main mediator of eclampsia, phosphocholinated neurokinin B, on the immune system and circulation (106). The role of inflammasomes in the pathogenesis of preeclampsia in the presence of tuberculosis or HIV infection in pregnant women has been demonstrated by Phoswa et al. (105). Tuberculosis with HIV infection can induce prolonged activation of placental inflammasomes such as NLRP3, AIM2, and NEK7. As a consequence, this persistent activation may induce an enhanced inflammatory response in the placenta (12, 33, 106). Local inflammation may lead to microthrombosis, endothelial dysfunction and ischemia.

Against the background of increased systemic levels of proinflammatory cytokines and compensatory neurokinin response of the placenta, preeclampsia eventually develops. At the same time, the expression levels of immune checkpoint antigens are impaired during pre-eclampsia as well as in tuberculosis or HIV infection (106). Interestingly, in chronic experimental arthritis, the ability of *Mycobacteria* from the adjuvant used for its induction to stimulate neurokinin expression has been shown (106), although similar studies have not been performed in tuberculosis. In pulmonary tuberculosis, pathomorphologic changes in the placenta corresponding to the picture of cell-compensated placental insufficiency, with impaired angiogenesis and differentiation are naturally observed, which creates prerequisites for reciprocal hypersecretion of neurokinins and preeclampsia (107). Physiologic transplacental transfer of antimycobacterial antibodies of IgG class from mother to fetus, in turn, is disrupted in late gestosis (107). In other words, tuberculosis infection and preeclamptic conditions are mutually unfavorable.

T-lymphocyte checkpoints used for down-regulation of autoreactivity are essential for normal pregnancy, and the use of their immunotherapeutic inhibitors in oncology is complicated by pregnancy disorders. In women with preeclampsia, however, there is a significant decrease in CTLA-4, TIM-3, and LAG 3 expression, but also an increase in PD-1 expression in Treg cells. And in tuberculosis (as well as in HIV infection and their combination), all immune checkpoint proteins are hyperexpressed, which may be interpreted as manifestations of a counterimmune response on the pathogens against host immune system (105).

### 4.3 Sex hormones and immunoreactivity in pregnancy

It should be emphasized that the peculiarities of immunoneuroendocrine reactivity of pregnant women have multiple causes, and are not exhausted by the action of the above mentioned bioregulators inherent to pregnancy.

The very belonging to the female sex, irrespective of pregnancy, causes increased resistance to tuberculosis in adulthood compared to men, which has been proved epidemiologically and in animal experiments and is clearly associated with the oppose effect of androgens and estrogens on the immune system (108). But, the major steroid hormone of pregnancy is progesterone, although the production of estrogens (significant immune stimulators) also increases in pregnancy. According to data from India, progesterone production decreases in women of childbearing age with pulmonary tuberculosis (109), possibly due to the action of the above-mentioned mycobacterial antigonadotropic factors (82). Progesterone deficiency has also been found in the structure of hypogonadism in tuberculosis-affected Nigerian women, going away with antituberculosis treatment (110). Progesterone is considered to be an immunosuppressive factor, although, on the contrary, its action is stimulatory with respect to the efficacy of IL-23 and IL-17A production, which are very essential for a successful antituberculosis response (111).

As for estrogens, although their physiological concentrations (outside of pregnancy) are considered to be a significant factor of increased resistance to tuberculosis in women compared to men (104), but it should be noted that in high concentrations peculiar to pregnancy they shift the vector of differentiation of T-helpers to the Th2-side, which cannot be considered favorable for antituberculosis resistance based mainly on the Th1-dependent response (112). Physiologic concentrations of estrogens suppress the production of anti-inflammatory and immunosuppressant cytokine IL-10 in rats, but high concentrations, similar to those created during pregnancy, on the contrary, stimulate it (113).

### 4.4 Prolactin and immune system in pregnancy

The prolactin, well-known natural contraceptive factor in pregnant and lactating women, is a stimulator of immunity and autoimmune responses, as well as macrophage function and antigen presentation, and acts with endo-, para- and juxtacrine manner, both as a hormone and as a cytokine, since it is produced not only by endocrinocytes but also by immunocompetent cells (114).

The tendency to hyperprolactinemia, which exists in chronic pulmonary granulomatoses (both in sarcoidosis and tuberculosis) has been reported by us and other researchers (115, 116). It is likely to be pathogenetically important. On the one hand, prolactin is an immunostimulant, and its action involves natural killer cells, participants of chronic granulomatous inflammation, whose role is essential in anti-mycobacterial defense. On the other hand, prolactin is an activator of autoimmune processes and can enhance autoimmunity in parallel with chronic granulomatous inflammation (117). Increased cytotoxic lymphocyte activity in hyperprolactinemia may contribute to miscarriages (54). Mycobacterial proteins in experiment strongly stimulate paracrine prolactin production in macrophages, which may contribute to the mechanism of hyperprolactinemia in tuberculosis (118). In turn, prolactin significantly modulates the cytokine response in tuberculosis, increasing inflammatory activation of macrophages and promoting apoptotic processes in granulomas. It has a restraining effect on the production of a number of proinflammatory cytokines (TNFα, IL-1β, IL-12), but promotes the production of fibrogenic IL-10 (119). Prolactin also stimulates the hydroxylation of vitamin D, with its conversion to active calcitriol, the most important stimulator of cathelicidin expression. The later is a key defensin of the antituberculosis response (120–122). Thus, hyperprolactinemia in pregnant women cannot but influence the course of their tuberculosis.

All together the above mentioned neuroendocrine changes in the organism of a pregnant women are resulted in the difficulties of TB diagnosis establishment in this cohort. The pregnancy causes the complex of immunoneuroendocrine shifts decreasing the effectiveness of cellular immunity, which may cause a less favorable course of a TB infection. Many authors insist that in pregnancy: “for this reason, it is important to consider TB as a possible diagnosis and to initiate the corresponding diagnostic test battery in the presence of suggestive symptoms” (123).

## 5 Conclusion

Tuberculosis in pregnant women occurs with pronounced disorders of immune and neuroendocrine response. The immuno-neuroendocrine changes characteristic of pregnancy have a multifactorial effect on antituberculosis immunity and determine the specificity of the course of TB against the background of pregnancy. HIV infection poses a particular threat, exacerbating immune response disorders that affect the effectiveness of treatment and disease progression in general. Because of challenging TB specifics in pregnancy, “under no circumstances should the treatment be delayed” (124) for pregnant women with proven TB. Even more so, taking into account that for several effective anti-TB medicines both foetotoxicity and teratogenicity were proven to be unlikely (125, 126).
